# A Randomized Trial of a New Triple Drug Treatment for Lymphatic Filariasis

**DOI:** 10.1056/NEJMoa1706854

**Published:** 2018-11-09

**Authors:** Christopher L. King, James Suamani, Nelly Sanuku, Yao-Chieh Cheng, Samson Satofan, Brooke Mancuso, Leanne J. Robinson, Peter M. Siba, Gary J. Weil, James W. Kazura

**Affiliations:** Center for Global Health and Diseases, Case Western Reserve University School of Medicine, Cleveland, OH, USA; Veterans Affairs Medical Center, Cleveland, OH, USA; Papua New Guinea Institute of Medical Research, Papua New Guinea; Papua New Guinea Institute of Medical Research, Papua New Guinea; Center for Global Health and Diseases, Case Western Reserve University School of Medicine, Cleveland, OH, USA; Papua New Guinea Institute of Medical Research, Papua New Guinea; Center for Global Health and Diseases, Case Western Reserve University School of Medicine, Cleveland, OH, USA; Papua New Guinea Institute of Medical Research, Papua New Guinea; Papua New Guinea Institute of Medical Research, Papua New Guinea; Department of Medicine, Infectious Diseases Division, Washington University School of Medicine, St. Louis, USA; Center for Global Health and Diseases, Case Western Reserve University School of Medicine, Cleveland, OH, USA

## Abstract

**BACKGROUND:**

The recommended drug regimen for elimination of lymphatic filariasis outside sub-Saharan Africa is single dose of diethylcarbamazine (DEC) plus albendazole (ALB). Multiple annual treatments are required for elimination since this regimen does not sustainably reduce blood microfilaria (Mf) counts below the threshold required to interrupt transmission. This study tested the efficacy of a single dose of ivermectin (IVM) combined with DEC/ALB.

**METHODS:**

We conducted an open label, randomized clinical trial in which *Wuchereria bancrofti*-infected adults in Papua New Guinea were assigned to treatment with DEC/ALB once (n=61), DEC/ALB twice (n=61, baseline, 12 months) and IVM/DEC/ALB once (n=60). The primary outcome was complete clearance of blood Mf at 24 months.

**RESULTS:**

At 24 months 96% of participants treated with IVM/DEC/ALB were amicrofilaremic compared to 56% after single dose DEC/ALB (relative risk for Mf+ = 0.08 [0.02-0.34, 95% CI], p<0.001) and 75% after two annual doses of DEC/ALB (relative risk for Mf+ = 0.15 [0.03-0.62, 95% CI], p=0.009). Filarial antigen levels decreased markedly and to a similar degree after treatment with all 3 regimens. Moderate adverse events were more common after the triple than the two-drug regimen (27% vs. 5%, p<0.001). There were no serious adverse events.

**CONCLUSIONS:**

Treatment with a single dose of IVM/DEC/ALB was superior to a single dose or two annual doses of DEC/ALB for clearing Mf. There were no significant safety concerns. Widespread use of IVM/DEC/ALB could greatly accelerate elimination of lymphatic filariasis. (ClinicalTrials.gov, NCT01978771)

## INTRODUCTION

Lymphatic filariasis (LF), an infectious disease caused by mosquito-borne nematode parasites, is characterized by lymphedema of the extremities (“elephantiasis”), hydroceles and chronic disability. The life cycle of the parasite requires uptake of microfilariae (Mf) by mosquitoes with their blood meal and development of Mf in the mosquito to infective larvae that are the transmission stage for new infections in humans 1. The filarial species *Wuchereria bancrofti* and to a lesser extent, *Brugia* spp., infect more than 100 million people in 73 countries with another one billion at risk 2. The World Health Organization (WHO) has targeted LF for global elimination by 2020 by means of mass drug administration (MDA)^3^ that uses one of three anti-filarial drug regimens: i) DEC/ALB in LF endemic areas outside Africa and in countries within Africa that do not have onchocerciasis or loiasis, ii) IVM/ALB in African countries that have both LF and onchocerciasis iii) ALB alone in countries that have both LF and loiasis. MDA is intended to reduce the Mf reservoir below a level that is required to sustain transmission of the infection by mosquitoes. Because a single dose of these treatments fails to sterilize or kill all adult filarial worms and reduce the community Mf reservoir to sufficiently low levels 4-7, many rounds of MDA are required to interrupt transmission.^8^ Although this approach has successfully eliminated LF in some countries, a treatment that is more effective for killing or sterilizing adult worms could greatly accelerate efforts to eliminate LF by reducing the number of doses and annual cycles of MDA required to interrupt transmission.

We recently reported results of a small pilot study that compared the pharmacokinetics and efficacy of a single dose of co-administered IVM/DEC/ALB versus DEC/ALB for bancroftian filariasis in Papua New Guinea 9. Triple drug therapy achieved 100% clearance of Mf at 12 and 24 months after treatment compared to 8% clearance after DEC/ALB, suggesting that IVM/DEC/ALB may have killed or permanently sterilized adult filarial worms. There were no severe or serious adverse events (AEs). The current randomized clinical trial aimed to evaluate the triple drug treatment compared to the standard DEC/ALB in a larger number of infected adult residents of an area of Papua New Guinea where LF is highly endemic and associated with high Mf burdens 4. 

## METHODS

### STUDY DESIGN AND PARTICIPANTS

A randomized, controlled, study was performed with participants recruited from 12 villages in Dreikikir district, East Sepik Province, Papua New Guinea. None of the participants had received previous treatment for LF. Institutional Review Boards at University Hospitals Cleveland Medical Center, Cleveland, OH (#04-12-33) and the Papua New Guinea Institute of Medical Research (#1220) and Medical Research Advisory Committee (#12.35) of Papua New Guinea approved the study protocols and documents. All participants provided written informed consent.

**Figure 1.**
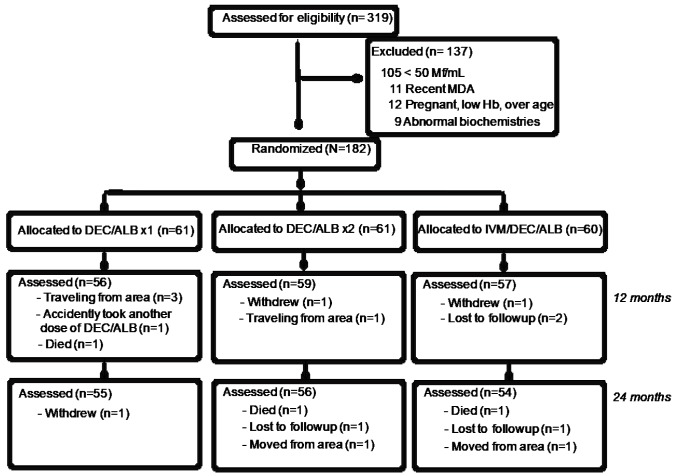
Enrollment and follow-up of participants in the treatment trial.

319 circulating filarial antigen (CFA) test positive individuals were screened for blood Mf levels. 182 participants met the inclusion criteria of >50 Mf/mL, age 18-65 years, no recent illness by history, non-pregnant, no prior treatment with DEC or ALB, no significant biochemical or hematologic abnormalities and no significant proteinuria, hematuria or glucosuria (Figure 1).

### RANDOMIZATION AND BLINDING

Eligible and consenting participants were randomized 1:1:1 to one of three treatment arms using a computer-generated randomization table: i) DEC 6mg/kg (Sanofi S.A., Gentilly, France) + ALB 400mg (GlaxoSmithKline, Uxbridge, United Kingdom) administered once at study initiation; ii) DEC 6mg/kg + ALB 400mg at study initiation and at 12 months, and iii) IVM 200 µg/kg (Merck & Co., Inc., Kenilworth, NJ, USA) + DEC 6mg/kg + ALB 400mg once at study initiation. A designated individual administered medications under direct observation to assure all pills were swallowed. Participants were unaware of the treatment arm. Blood samples were labelled only with ID numbers. Individuals counting Mf and evaluating adverse events (AEs) were blinded to treatment assignment. 

### PROCEDURES

Screening and initial treatment were performed at the Dreikikir health center under direct observation for ten hours and monitored for AEs over the next two days. A symptom questionnaire was administered and vital signs obtained and repeated after treatment. A symptom-directed physical examination was performed if moderate or severe subjective AEs were reported. New or worsening symptoms, changes in vital signs and new abnormal physical examination findings were considered to be drug-related AEs and scored using a modified version of the National Cancer Institute Common Terminology Criteria for Adverse Events, v4.0.

Microfilaremia was assessed by passing two mL of heparinized blood (collected by venipuncture between 9 p.m. and 1 a.m.) through two 5 μm polycarbonate filters (one mL per filter, EMD Millipore Corp). Filters were washed, placed on glass slides, dried, stained with Giemsa and read by microscopy for the presence of Mf as previously described 10.

Circulating filarial antigen levels were measured by ELISA at baseline, 12 and 24 months, as previously described 11,12. Analysis was limited to participants with CFA levels above 15ng/mL at baseline and for whom samples were available for all time points (N=48,45,42 for the DEC/ALBx1, DEC/ALBx2, IVM/DEC/ALBx1 arms respectively). Individuals with lower baseline CFA levels were excluded from the percent reduction analysis since measurement of CFA levels is not accurate near the lower limit of detection (6.8 ng/mL). 

### OUTCOMES

The primary outcome was percent of individuals with total Mf clearance at 24 months post- treatment. Secondary outcomes were percent Mf clearance at 12 months, reduction in Mf counts, percent of individuals who cleared CFA and percent reduction in CFA relative to baseline.

### STATISTICAL ANALYSIS

The primary hypothesis was that IVM/DEC/ALB given once would achieve 75% Mf clearance at 36 months compared to 50% Mf clearance with a single dose DEC/ALB. A second hypothesis is that single dose of IVM/DEC/ALB would be non-inferior to DEC/ALB given annually with a confidence margin of 15%. For alpha=0.05 and power of 0.8 we estimated 46 and 54 individuals would be required for each arm to test the first and second hypotheses respectively. Additional participants were recruited to account for potential dropout. We conducted an unplanned 24- month interim analysis based on the unexpectedly high efficacy of IVM/DEC/ALB at 24 months observed in a separate pilot study 9. Because of the greater efficacy of IVM/DEC/ALB and the potential importance of these results for the Global Programme to Eliminate Lymphatic Filariasis, we decided to report the results of the 24-month interim analysis based on recommendations of our technical advisory committee. We were also concerned about risk of re-infection in study participants that resided in communities where MDA for LF had been delayed. We performed an intent-to-treat (ITT) analysis for all individuals for whom a sample was collected at 24 months. Mf counts were expressed as Mf/mL+1 and log transformed; geometric mean values (GM) were used as measures of central tendency. Baseline characteristics and Mf clearance rates by treatment group as well as differences in Mf counts and circulating antigen levels at 12 and 24 months after treatment relative to baseline were compared using the chi-squared test and the Kruskal-Wallis H test. A generalized estimating equation (SAS v 9.2) compared the Mf clearance relative to baseline among treatment arms and evaluated the independent effects of age, sex, baseline Mf counts, and village location on Mf clearance rate. 

### ROLE OF THE FUNDING SOURCE

The study sponsor had no role in study design, data collection, analysis, interpretation, or writing of the report.

## RESULTS

### ENROLLMENT AND FOLLOW-UP 

Participants were enrolled between June 11 and December 13, 2014. Baseline demographics, Mf counts, and CFA levels were similar among the three treatment groups (Table 1). Pretreatment CFA levels correlated positively with pre-treatment Mf counts (Spearman’s rho: 0.42, p=0.02). 95% and 91% of subjects were available for follow-up at 12 and 24 months post- treatment. Reasons for loss-to-follow-up are shown in Figure 1. Three participants died from causes unrelated to the study. These were probable liver cancer (DEC/ALB x 1), snakebite (IVM/DEC/ALB), and probable suicide (DEC/ALB x 2). 

**Table 1:** Characteristics of Study Participants at Baseline

	Treatment Groups		
	DEC/ALB x 1	DEC/ALB x 2	IVM/DEC/ALB x 1
N	61	61	60
Age Median (range)	34 (18-62)	37 (18-61)	40 (19-60)
Sex (M/F)	34/27	30/31	28/32
Hemoglobin gm/dL (mean ± SD)	11.2 (1.8)	11.2 (1.7)	11.4 (1.8)
Weight kg (mean ± SD)	51 (5)	52 (7)	50 (6)
Microfilaria/mL geomean (Range)	744 (52-8,290)	596 (61-9,656)	699 (55-15,621)
Circulating Filarial Antigen ng/mL geomean (Range)	79 (18-340)	81 (15-325)	72 (17-348)

DEC – diethylcarbamazine, ALB – albendazole, IVM – ivermectin

### EFFECTS OF TREATMENT ON MICROFILAREMIA

**Figure 2.**
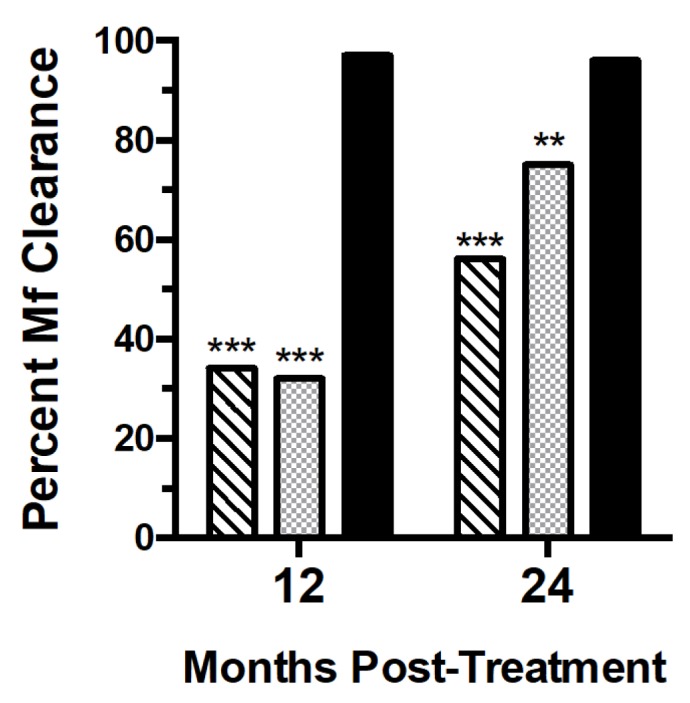
Percent of participants with complete Mf clearance at 12 and 24 months post- treatment with DEC/ALB x 1 (hatched bars), DEC/ALB x 2 (light bars), and IVM/DEC/ALB x 1 (dark solid bars). Mf clearance rates were significantly higher for the IVM/DEC/ALB x 1 group compared to the other two groups at both 12 and 24 months (***p<0.001, chi-square). Complete Mf clearance at 24 months was more common in the DEC/ALB x 2 group compared to DEC/ALB x 1, p=0.004.

With respect to the primary outcome at 24 months (Figure 2), a single dose of IVM/DEC/ALB at baseline completely cleared Mf in 52 of 54 participants (96%, [95% CI, 92%, 100%]) compared to 31 of 55 participants (56% [46%, 66%]) treated with a single dose of DEC/ALB once at baseline (relative risk was 0.08 (95% CI, 0.02-0.34, p<0.001). DEC/ALB administered twice (at baseline and 12 months post-treatment) cleared blood Mf in 42 of 56 participants (75%, 95% CI 67%, 83%). This clearance rate was significantly lower than that after a single dose of IVM/DEC/ALB (relative risk was 0.15 (0.03-0.62, 95% CI, p=0.009). At 12 months IVM/DEC/ALB completely cleared Mf in 57 of 59 participants (96%, [95% CI, 92%, 100%]) compared to 18 of 56 participants (32% [22%, 41%]) and 20 of 59 (34% [25%, 43%]) following a single dose of DEC/ALB at baseline in the two other treatment arms. There were significant differences in Mf clearance by treatment group at 24 months using a generalized estimating equation adjusted for location, age, sex and pretreatment Mf levels (odds ratios of 46 and 30 relative to DEC/ALBx1 and DEC/ALBx2 respectively, p<0.0001, Table S1). In this model, higher pre-treatment Mf counts were associated with 3% reduced likelihood of completely clearing Mf at 24 months (p=0.004). Mf clearance was not significantly associated with age or village of residence, however women were 52% more likely to be Mf negative compared to men (p=0.014). 

With respect to missing data, if we assumed all missing participants in the IVM/DEC/ALB arm were Mf positive (complete clearance in 54 of 60 participants, 90%) and that all individual missed in the two DEC/ALB arms were Mf negative (complete clearance in 36 of 61 participants [59%] after DEC/ALBx1 and in 47 of 61 [77%] after DEC/ALBx2), IVM/DEC/ALB would still have had significantly higher Mf clearance rates at 24 months than either DEC/ALB treatment arms (p<0.001. p=0.04, respectively, chi-square).

**Figure 3.**
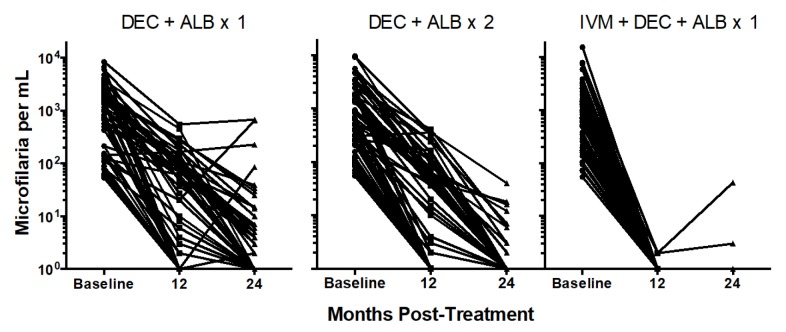
Reductions in Mf counts at 12 and 24 months post-treatment. Note the log scale + 1 for Mf counts. A single dose of IVM/DEC/ALB was significantly more effective for reducing Mf counts than either of the two DEC/ALB treatments at 12 and 24 months, p<0.001, Mann- Whitney U test. The DEC/ALB x 2 group had greater reductions in Mf counts than the DEC/ALB x 1 group at 24 months, p=0.004.

The geometric mean (GM) Mf count in individuals with persistent Mf at 24 months following a single dose of DEC/ALB was 12 Mf/mL (range 1-671); four participants of the total number of persons tested at 24 months (7%) had >50 Mf/mL (Figure 3). The GM Mf count in individuals with persistent Mf at 24 months following two annual doses of DEC/ALB was 5 Mf/mL (range 1-39). At 24 months post IVM/DEC/ALB, the two Mf+ participants, one with 2 Mf/mL and the other with 44 Mf/mL (Figure 3). The same two individuals were Mf positive at 12 months with 1 Mf/ml each. 

### EFFECTS OF TREATMENT ON CIRCULATING FILARIAL ANTIGENEMIA

CFA levels decreased significantly by 12 months after treatment relative to baseline in all treatment groups with further reductions between 12 and 24 months. The DEC/ALB once and DEC/ALB twice treatment groups had similar reductions in CFA levels of 58%-59% and 70%- 71% at 12 and 24 months, respectively. These values were less than the 67% and 75% reductions observed at 12 and 24 months after IVM/DEC/ALB treatment, but the differences were not statistically significant. More people in the IVM/DEC/ALB treatment arm had CFA levels reduced to below the assay’s limit of detection at 24 months (14 of 42 or 32%) than those in the other treatment groups (10 of 48 or 21% in the DEC/ALB x 1 group and 11 of 45 or 24% in the DEC/ALB x 2 treatment group), but these differences were not significant. Relative CFA levels after treatment were not significantly lower in individuals who completely cleared Mf after treatment compared to individuals with persistent Mf at 12 and 24 months (p = 0.07 and 0.30).

### SAFETY

All individuals were actively observed for up to 10 hours post-treatment for AE, and 73% of participants were assessed for AEs between 24 to 36 hours after returning to their villages (Table 2). Five participants experienced AEs during the initial 10 hours observation period; three AEs were mild, one was moderate, and one was severe. The individual with a severe AE was a 42-year-old woman with a pre-treatment Mf count of 792/mL who experienced headache, nausea and chills starting six hours after taking IVM/DEC/ALB. Physical examination revealed a temperature of 41.1°C, orthostatic hypotension, and tachycardia. She was successfully treated with oral fluids and acetaminophen and returned to her pre-treatment state of health the following day. Objective findings of fever (temperature >37.8°C) and hemodynamic changes following initial treatment tended to be higher in participants receiving IVM/DEC/ALB; however, these differences were not significant (Table 2). The frequency of subjective AEs was greater in participants who received triple drug treatment versus DEC/ALB. The difference was most pronounced in individuals who had AEs with severity >1. The most common AEs reported were headache, fatigue and nausea both for persons with grade 1 AEs (data not shown) and for persons who experienced AEs with severity >1 (Table 2). Higher pre-treatment Mf counts were associated with a greater frequency and severity of AEs. Using a logistic regression model, the odds of a grade 2 AE increased by 19% for each 200 increase in Mf/mL count (OR 1.19 [95% CI 1.09,1.36], p=0.01). This association was greatest among individuals with >500 Mf/mL. Of note, no participant experienced an AE with severity greater than grade 1 and none had fever or changes in blood pressure after their second dose of DEC/ALB. Consequently, only AEs associated with the initial treatment are included in Table 2, and AEs for the two DEC/ALB treatment arms at baseline are combined. 

**Table 2:** Adverse Events (AEs) Following Treatment for Lymphatic Filariasis

	DEC/ALB (N=91)	IVM/DEC/ALB (N=41)
	Number of participants with AEs (percent)	
At least one AE	37 (41)	24 (59)
Individuals with two or more AEs	24 (26)	19 (46)*
Fever§	19 (21)	14 (34)
Hemodynamic changes¶	4 (4)	5 (12)
Overall Grade 1 AEs (subjective)†	36 (40)	22 (54)
Overall Grade 2 and 3 AEs with severity grade >1†	5 (5)	11 (27)***
Frequency of AEs with severity grade >1		
Fatigue	5 (5)	8 (20)
Headache	3 (3)	7 (17)
Nausea/vomiting	2	4 (10)
Itch/rash	0	2 (5)
Muscle ache	3 (3)	5 (12)
Eye swelling	0	1 (2)
Scrotal pain/swelling	2 (2)	4 (10)
Dyspnea	0	2 (5)

* p<0.05

§ Auricular temperature ≥37.5°C. The highest temperature recorded post-treatment was 41.1°C.

¶ Defined as a change in blood pressure of 30 mm Hg systolic or 20 mm Hg diastolic compared to the pre-treatment recording. Three of five individuals in the IVM+DEC+ALB group had reduced blood pressure. All four individuals in the DEC+ALB group had reduced blood pressure.

† Only grade 2 or 3 AEs are listed by symptoms. All but one participant with grade 2 symptoms had more than one AE.

*** p<0.001 by chi-square

## DISCUSSION

These results show that a single dose of the new triple drug regimen consisting of IVM/DEC/ALB was much more effective for clearing *W. bancrofti* blood Mf than treatment with standard MDA of DEC/ALB in LF-endemic areas outside of sub-Saharan Africa. Participants in this clinical trial had not been previously treated for LF, and they had moderate to very high blood levels of *W. bancrofti* Mf and filarial antigenemia. A single dose of triple drug therapy cleared Mf from almost all participants, and the effects persisted for at least 24 months. This regimen was superior for clearing Mf compared to a single dose or two annual doses of DEC/ALB. Results observed after DEC/ALB treatment were consistent with those reported from previous trials 9,13. Although a single dose of triple therapy did not completely clear Mf in every subject, residual Mf counts were reduced to levels unlikely to support mosquito-borne transmission 14,15. Both IVM/DEC/ALB and the two drug regimen of DEC/ALB had potent macrofilaricidial effects based on >70% reductions in CFA levels 24 months after treatment commenced. These data are consistent with prior studies that documented partial macrofilaricidal effects of DEC/ALB 16,17,18, whereas IVM has little or no ability to kill adult worms 19. The addition of IVM to DEC/ALB had only a marginal impact on reducing CFA levels, but the triple drug combination appears to be very effective for sterilizing adult worms, an effect that may be permanent based on observations to date. Limitations are that an open-labeled study could bias assessment of adverse events and blood Mf detected at the time of follow-up could be due to reinfection in some cases. However, we believe this is unlikely because of high rates of bed net use in study communities. 

Adverse events were more frequent after treatment with IVM/DEC/ALB. This result is consistent with observations in a pilot study performed by our group 9. Since AEs are triggered by Mf death, it is not surprising that AEs were more common in people treated with two potent microfilaricidal drugs (DEC/IVM). The single severe AE that occurred was self-limited, similar to AEs reported in earlier studies after individuals were treated with DEC/IVM or DEC alone 20. AE frequency and severity after triple drug treatment are likely to be much lower in community MDA settings where infection rates and blood Mf levels are lower than those in this clinical trial. Indeed, a recently completed, multicenter community safety study performed in LF-endemic areas found the same rates and severity of AEs after MDA with either IVM/DEC/ALB or DEC/ALB (authors’ unpublished data). 

Simulation-modeling studies suggest that MDA with IVM/DEC/ALB should significantly reduce the number of rounds of MDA required to reach elimination targets 21. Thus, triple drug MDA with IVER/DEC/ALB provides a potential road to success for countries that are not currently on track to eliminate LF by the current target year of 2020 2,22. 

## Supplementary Materials

Supplementary Materials
