# Supplementary material for: A Randomized Trial of a New Triple Drug Treatment for Lymphatic Filariasis
Source: N Engl J Med. Author manuscript; Available in PMC 2018 Nov 9. (PMC6194477; doi:10.1056/NEJMoa1706854)
Supplement: Supplementary Materials [file NEJMoa1706854_King_Supplement.pdf]

Appendix - Supplementary Information

Table of Contents

Page 1. Authors contributions

Page 2. Table S1. Output of generalized estimating equation.

Methods

Authors roles: Designed study (CLK, JWK, GJW), gathered data (JKS, NS, Y-CC, BM, CLK), coordinated the study (CLK, LJR, PMS, BM, Y-CC), analyzed the data (CLK, Y-CC, GJW, JWK), and CLK vouches for the data and analysis. GJW, CLK and JWK decided to publish. CLK wrote the first draft, revised by GJW and JWK, and all authors contributed to writing subsequent drafts.

Table S1. The odds of complete clearance of blood Mf with IVM/DEC/ALB x 1 relative to DEC/ALB x 1 and DEC/ALB x 2 at 24 months post-treatment adjusted for age, sex, village of residence and baseline Mf levels at using a generalized linear model.

**Tmt**	**Odds ratio**	**SE**	**95% CI**		**P**
DEC/ALB x 2	30.3	2.4	5.6	162.6	<*0.0001*
DEC/ALB x 1	46.2	2.4	8.5	252.1	<*0.0001*
IVM/DEC/ALB x 1	reference				
Age	0.99	1.01	0.97	1.02	0.628
Sex (F relative to M)	0.48	1.35	0.26	0.86	*0.014*
Village	0.92	1.12	0.87	1.14	0.452
Mf Baseline	1.03	1.01	1.01	1.05	*0.004*

- Analysis was performed using a generalized estimating equation (GEE, SAS v 9.2)
- Quasi Likelihood under Independence Model Criterion (QIC) was 335 suggesting a good model fit.
- The independent effects of age, sex, location and baseline Mf are shown.
